# The RECIPE study: reducing emergency Caesareans and improving the Primiparous experience: a blinded, prospective, observational study

**DOI:** 10.1186/s12884-020-03112-6

**Published:** 2020-07-29

**Authors:** Niamh C Murphy, Naomi Burke, Patrick Dicker, Fiona Cody, Etaoin Kent, Elizabeth C Tully, Fergal D Malone, Fionnuala M Breathnach

**Affiliations:** 1 Obstetrics & Gynaecology, Royal College of Surgeons in Ireland, Dublin, Ireland; 2 Epidemiology & Public Health, Royal College of Surgeons in Ireland, Dublin, Ireland; 3 Obstetrics & Gynaecology, Rotunda Hospital, Dublin, Ireland

**Keywords:** Caesarean delivery, Risk prediction, Model validation, Risk stratification, Personalised care

## Abstract

**Background:**

The RECIPE study aims to validate a risk prediction model for intrapartum caesarean delivery which has been developed by our group. The Genesis study was a prospective observational study carried out by the Perinatal Ireland Research Consortium across 7 clinical centres in Ireland between October 2012 and June 2015. Genesis investigated a range of maternal and fetal parameters in a prospective blinded study of 2336 singleton pregnancies between 39 + 0–41 + 0 weeks’ gestational age. This resulted in the development of a risk prediction model for Caesarean Delivery in nulliparous women at term. The RECIPE study now proposes to provide external validation of this risk prediction tool.

**Methods:**

In order to externally validate the model, we aim to include a centre which was not involved in the original study. We propose a trial of risk-assignment for intrapartum caesarean amongst nulliparous women with a singleton pregnancy between 38 + 0 and 40 + 6 weeks’ gestational age who are planning a vaginal birth. Results of the risk prediction tool will be concealed from participants and from midwives and doctors providing labour care.. Participants will be invited for an ultrasound scan and delivery details will be collated postnatally. The principal aim of this study is to externally validate the risk prediction model. This prediction model holds the potential to accurately identify nulliparous women who are likely to achieve an uncomplicated vaginal birth and those at high prospect of requiring an unplanned caesarean delivery.

**Discussion:**

Validation of the Genesis prediction model would enable more accurate counselling for women in the antenatal setting regarding their own likelihood of requiring an intrapartum Caesarean section. It would also provide valuable personalised information to women about the anticipated course of their own labour. We believe that this is an issue of national relevance that will impact positively on obstetric practice, and will positively empower women to make considered, personalised choices surrounding labour and delivery.

## Background

The OECD reports that Ireland has a Caesarean section rate of 28.5 per 100 live births [1].

The Irish National Maternity Strategy has highlighted the escalating Caesarean section rate as an area for concern and has identified Caesarean delivery as a key focus for change over the next ten years [2]. Of note, the Strategy also highlights the importance of reducing the rate of intrapartum caesarean delivery, owing to the heightened degree of both maternal and perinatal morbidity associated with unplanned Caesarean delivery performed during the course of labour. Postoperative complications, including haemorrhage and perioperative infection in women who undergo unplanned Caesarean delivery are significantly higher when compared to women who undergo elective Caesarean delivery.

Genesis [3] was a prospective, observational study carried out across seven clinical sites within the Perinatal Ireland Research consortium.

A total of 2336 nulliparous women were recruited to the study and underwent assessment at 39–41 weeks gestation that included an ultrasound scan and documentation of fetal and maternal anthropometric data. Delivery outcomes were subsequently collated.

Among 37 potential maternal, fetal, sociodemographic and obstetric factors studied, Genesis identified five key parameters (advancing maternal age, shorter maternal height, increasing BMI, larger fetal abdominal circumference and larger fetal head circumference) that represented the optimal prenatal predictive factors for intrapartum Caesarean section delivery in a composite model. A resultant algorithm was developed (the Genesis Risk Prediction Tool) as a means of providing a personalised risk assessment for Caesarean delivery in this population of nulliparous women with singleton uncomplicated pregnancies who were planning a vaginal birth [3]. The aim of the RECIPE study is to validate this prediction tool in a new population of women and to expand recruitment to include women from 38 + 0–41 + 0 weeks gestation. RECIPE will also recruit women from a new hospital site and include women who have pre-existing medical conditions which were excluded from Genesis.

## Methods/design

The primary objective of the RECIPE study is to externally validate the Genesis predictive risk tool to examine if it can accurately predict the need for emergency caesarean section delivery in nulliparous women.

Secondary objectives include validation in an extended obstetric population, to enumerate maternal or neonatal complications and to ascertain women’s satisfaction with their labour experience with a postnatal interview.

### Study design

This is a multicentre, blinded prospective observational study. All nulliparous singleton pregnancies with a cephalic presentation will be considered for enrolment. Exclusion criteria include women with a plan for caesarean delivery, multiple pregnancy, non-vertex presentation, known major fetal abnormality, uncertain gestational age, rupture of membranes or ultrasound-indicated reason for delivery at the time of ultrasound scan.

The study will be carried out in two clinical research sites-the Rotunda Hospital, Dublin and Our Lady of Lourdes Hospital, Drogheda. Recruitment will take place in the Rotunda Hospital Dublin from May 2017–March 2019 and in Our Lady of Lourdes Hospital, Drogheda from May 2018–March 2019. Both centres have tertiary-level neonatology facilities and the latter site includes a midwifery-led delivery unit.

### Study procedures

#### Antenatal clinical assessment and ultrasound scan

Maternal demographic and anthropometric details will be collected at the scheduled appointment with the research sonographer.

A fetal ultrasound scan will be performed to obtain fetal biometry at a gestational age of 38 + 0–41 + 0 weeks.

Fetal biometry will include the following parameters: Fetal Head Circumference (HC), Bi-Parietal Diameter (BPD), Abdominal Circumference (AC) and Femur Length (FL). The Estimated Fetal Weight (EFW) will be obtained using the Hadlock-4 formula (HC, BPD, AC, FL). EFW will be freely available to women and clinicians and this differs from the Genesis study where these details were blinded in order to reduce bias.

Fetal biometry and amniotic fluid volume will be reported to the patient and care providers, however the individualized calculated risk for requiring an intrapartum cesarean delivery will be determined post-patient encounter/ ultrasound and will be concealed from the participants, midwives and clinicians..

In the event of an abnormal scan finding, the ultrasonographer will contact the patient’s obstetric team and that woman will be excluded from the study.

Abnormal scan findings which would necessitate such exclusion include the following: Ultrasound-indication for delivery at time of recruitment scan (Fetal growth restriction <3rd percentile, biophysical profile </=4/8, oligohydramnios DVP (Deepest Vertical Pool) < 2 cm).

### Intrapartum and neonatal details

Delivery outcome data will be collated by the research team within 42 days of delivery.

### Postnatal interview

Women participating in the study will be contacted by a member of the research team at approximately three months postpartum. This will be conducted as a telephone interview. The aim of this postnatal interview is to ascertain if the women had any complications following discharge from hospital.

### Sample size calculation

Collins et al. have suggested that externally validating a prognostic model requires a minimum of 100 events coinciding with other approaches to externally validating predictive models for binary events [4]. Using this rationale, the sample size required is 500 nulliparous women, assuming a 20% intrapartum Caesarean section rate in the study population. The original Genesis study had a 21% intrapartum Caesarean section rate, with a similar cohort [3].

### Data collection and analysis

Data management, data quality checks and statistical analysis will be performed by the trial biostatistician. The database will be anonymized, encrypted and stored in accordance with data protection law. Patient details will be recorded under an assigned study number, and access to the database will be password-protected to ensure access only by the study investigators.

The study will be analysed as a cohort to assess the accuracy of the Genesis predictive tool at identifying the women who will most likely require an intrapartum caesarean delivery as well as further analysis of the secondary objectives. The prediction model developed in the Genesis Study utilized a low-risk population, with a blinded third trimester ultrasound exam [3]. The exclusion criteria included: multiple pregnancy, non-vertex presentation, multiparous women, gestational age < 39 weeks, fetal abnormalities or aneuploidies, uncertain dates, pregnancy complications (e.g. growth restriction, pre-eclampsia) and pre-existing medical conditions (e.g. diabetes) and those considered for an elective caesarean section.

The Genesis prediction model [3] incorporated a third trimester EFW. Ultrasound EFW was accurate to within 10% of birthweight in 759 (87%) of patients and within 5% of birthweight in 428 (49%) of patients within 1 week of the delivery date.

This study is an external validation (specifically, spectrum transportability) of the prediction model in a population without a blinded third-trimester scan and who may be at an elevated risk for emergency cesarean section, compared to the original Genesis population. Model calibration and discriminative ability (e.g. AUC) will be assessed in this population. However, it is known that prediction models fitted with logistic regression often show poor performance when applied in populations other than the development population [5]. Since model updating may improve predictions, a closed-testing procedure will be used to update the prediction model [6]. This method balances the amount of evidence for updating the model in the new patient sample against the danger of overfitting.

The statistical packages SAS Version 9.4 and Stata Version 15 will be used to screen for outliers and anomalous values, tabulate, graphically summarize and model the data.

**Fig. 1:**
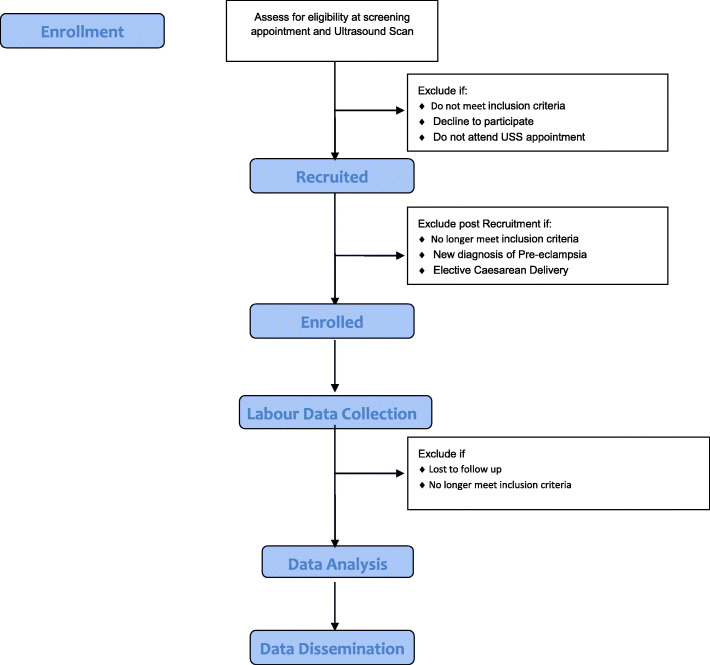
CONSORT Flow Diagram for RECIPE Study

A CONSORT flow diagram can be viewed at Fig. 1.

### Equipment and resources

The ultrasonography departments at the Rotunda Hospital and at Our Lady of Lourdes both have high quality imaging equipment alongside computer hardware and GE Viewpoint image analysis software. A research sonographer in the Rotunda hospital will perform the majority of the ultrasound scans in this unit, along with a senior registrar who is co-ordinating the study. A senior registrar in Our Lady of Lourdes hospital will perform the ultrasound scans and will undergo training with the lead research ultrasonographer in the Rotunda to ensure the highest quality images are being recorded. All images will be periodically reviewed by the research sonographer for quality assurance purposes.

## Discussion

If achievable, the ability to predict the outcome of an attempt at first labour is highly desirable. This prognostic model has been devised to assist clinicians and patients in individualized decision-making that will result in the safest mode of delivery for a first-time mother. At present, we are faced with a knowledge gap. We have devised a tool which can offer a risk prediction for intrapartum caesarean section delivery in low-risk nulliparous women. However, this tool requires careful validation in an expanded setting before it can be tested in a randomized trial and ultimately integrated into clinical practice.

Predictive and prognostic tools are being developed and utilised in other fields of medicine. Various modelling tools have been developed to address the lack of standardised processes that incorporate the perspective of all healthcare stakeholders. Such models can assist us in the decision-making process aimed at achieving specific clinical outcomes (in this instance, an accurate prediction of the likelihood of requiring an intrapartum Caesarean delivery).

The aim of the RECIPE study is to validate the Genesis risk prediction tool.

Historically, the field of obstetrics has been successful in developing predictive models but has been poor in validation and implementation [7]. A validated and accurate prediction tool for intrapartum Caesarean delivery holds far-reaching potential individual, health policy and societal benefit in a clinical space that is fraught with unpredictability. Ultimately, validation should enable our research group to design a randomized controlled trial of revealed versus concealed risk-prediction, with maternal morbidity as the proposed primary outcome. Furthermore, the knowledge of a high likelihood of a successful vaginal delivery has been shown to be as significant motivating factor for women entering labour [8]. This knowledge would be welcomed by the women we are caring for as they prepare for their upcoming labour.

## Data Availability

Not applicable.

## Abbreviations

RECIPE study: Reducing Emergency Caesareans and Improving Primiparous Experience
OECD: Organisation for Economic Co-Operation and Development
HC: Fetal Head Circumference
BPD: Bi-Parietal Diameter
AC: Abdominal Circumference
FL: Femur Length
EFW: Estimated Fetal Weight
DVP: Deepest Vertical Pool
